# Requirement of NOX2 and Reactive Oxygen Species for Efficient RIG-I-Mediated Antiviral Response through Regulation of MAVS Expression

**DOI:** 10.1371/journal.ppat.1000930

**Published:** 2010-06-03

**Authors:** Anton Soucy-Faulkner, Espérance Mukawera, Karin Fink, Alexis Martel, Loubna Jouan, Yves Nzengue, Daniel Lamarre, Christine Vande Velde, Nathalie Grandvaux

**Affiliations:** 1 CRCHUM - Centre Hospitalier de l'Université de Montréal, Montréal, Québec, Canada; 2 Department of Biochemistry, Faculty of Medicine, Université de Montréal, Montréal, Québec, Canada; 3 Department of Medicine, Faculty of Medicine, Université de Montréal, Montréal, Québec, Canada; North Carolina State University, United States of America

## Abstract

The innate immune response is essential to the host defense against viruses, through restriction of virus replication and coordination of the adaptive immune response. Induction of antiviral genes is a tightly regulated process initiated mainly through sensing of invading virus nucleic acids in the cytoplasm by RIG-I like helicases, RIG-I or Mda5, which transmit the signal through a common mitochondria-associated adaptor, MAVS. Although major breakthroughs have recently been made, much remains unknown about the mechanisms that translate virus recognition into antiviral genes expression. Beside the reputed detrimental role, reactive oxygen species (ROS) act as modulators of cellular signaling and gene regulation. NADPH oxidase (NOX) enzymes are a main source of deliberate cellular ROS production. Here, we found that NOX2 and ROS are required for the host cell to trigger an efficient RIG-I-mediated IRF-3 activation and downstream antiviral *IFNβ* and *IFIT1* gene expression. Additionally, we provide evidence that NOX2 is critical for the expression of the central mitochondria-associated adaptor MAVS. Taken together these data reveal a new facet to the regulation of the innate host defense against viruses through the identification of an unrecognized role of NOX2 and ROS.

## Author Summary

The understanding of the mechanisms allowing the host to mount a rapid and efficient innate immune response to RNA viruses has been the subject of intensive research in recent years. Major groundwork allowed the identification of key sensors of virus nucleic acids, including RIG-I and Mda5, which through association with the MAVS adaptor initiate the signaling cascade required for activation of the IRF-3 transcription factor and downstream antiviral genes. Mechanisms of activation and degradation of key signaling molecules allow a tight control of the intensity and duration of the response. Our knowledge of how redox processes regulate signaling cascades is a fast moving field of research. Particularly, the identification of non-phagocytic reactive oxygen species-producing NADPH oxidase (NOX) enzymes revealed new insights into their function in innate immunity. Our endeavor in characterizing the role of NOX in the antiviral response reveals a new facet to the overall picture of antiviral response regulation. Here, we demonstrate that NOX2 is essential for MAVS expression in airway epithelial cells, thereby controlling the capacity of the cell to mount an efficient innate antiviral response following recognition of viruses.

## Introduction

The capacity of the host to rapidly respond to virus infection is essential to establish an antiviral state that restricts virus replication and spreading, and to permit the production of proinflammatory chemokines and cytokines that attract and activate immune cells to the site of infection. Although major breakthroughs have recently been made, much remains unknown in our understanding of the molecular mechanisms involved in virus recognition and how it is transmitted via signaling messengers to the expression of antiviral and proinflammatory genes.

Initiation of these innate immune responses is achieved through recognition of invading viruses by pattern recognition receptors (PRR) that specifically recognize pathogen-associated molecular patterns (PAMPs). Virus-derived nucleic acids are considered major PAMPs that activate various PRRs, including members of the membrane-bound Toll-like receptors (TLRs) family, TLR-3, -7 and -9, and the recently identified cytoplasmic RIG-I-like receptors (RLRs), including RIG-I and Mda5 [1]. Following recognition of viral RNAs, RIG-I/Mda5 elicit signaling cascades via a caspase recruitment domain (CARD)-mediated interaction with the mitochondria-associated adaptor MAVS, also known as CARDIF/IPS-1/VISA [1], which in turn interacts with the TNFR-associated death domain (TRADD) protein [2]. At this level, the signaling cascades diverge due to specific interactions either with the FADD and RIP1 adaptors or with the E3 ubiquitin ligase TRAF3 and the adaptor protein TANK, to elicit activation of the NF-κB and IRF-3 transcription factors, respectively [2].

Activation of the ubiquitously expressed IRF-3 transcription factor is central to the development of an antiviral state, mainly through the rapid and robust expression of type I Interferons (IFNs) genes, a prerequisite for the induction of numerous antiviral proteins that modulate protein synthesis, growth arrest and apoptosis [3]. Moreover, IRF-3 also has the capacity to directly regulate a subset of these antiviral genes, including *IFIT1*, which encodes for the ISG56 translation regulator, thereby establishing an early IFN-independent antiviral response [4]. Virus-induced IRF-3 activation relies on a complex set of phosphorylation events mediated at least by the IκB-kinase (IKK)-related kinases, TANK binding kinase-1 (TBK1) and IKKε, that regulates its dimerization, nuclear accumulation and transactivation capacities [5], [6].

Reactive oxygen species (ROS), such as hydrogen peroxide and superoxide anion, are now well appreciated to act as cellular switches for signaling cascades leading to gene regulation involved in physiological processes, including cell proliferation, apoptosis and immune and proinflammatory responses [7], [8]. Amongst the enzymatic systems that produce ROS, the family of NADPH oxidase/Dual oxidase (NOX/DUOX) enzymes is now considered predominant in various cell types. Seven members of this family have been identified, named NOX1-5 and DUOX1-2, each with tissue- and cell-type specific expression patterns [9]. NOX2, which is mainly expressed in, but not restricted to, neutrophils and macrophages, is well known to play pivotal roles in host defense against bacterial and fungal pathogens, through production of superoxide in the phagosome [10]. Interestingly, recent functional data have emerged that suggest the involvement of NOX/DUOX members in the innate host defense to invading microorganisms in non-phagocytic cells [11], [12]. Particularly, a crucial role of NOX1 and NOX4 in the regulation of TLR-mediated intracellular signaling via MAPK and NF-κB has previously been highlighted [11], [13]. More recently, NOX2 interaction with TLR2 was shown to be required for efficient innate immune responses to Mycobacteria [14]. Additionally, we recently reported for the first time that NOX2 plays an essential role during *Paramyxoviridae* virus infections through the regulation of the NF-κB-mediated proinflammatory response in airway epithelial cells (AEC) [15]. Here, we add a new facet to the regulation of the antiviral response. Our data demonstrate that NOX2 and ROS are critical for the ability of the host cell to trigger an efficient RIG-I-mediated IRF-3 activation and downstream antiviral genes through regulation of MAVS expression.

## Results

### NADPH oxidase-derived ROS are required for SeV-induced antiviral genes regulation

**Figure 1:**
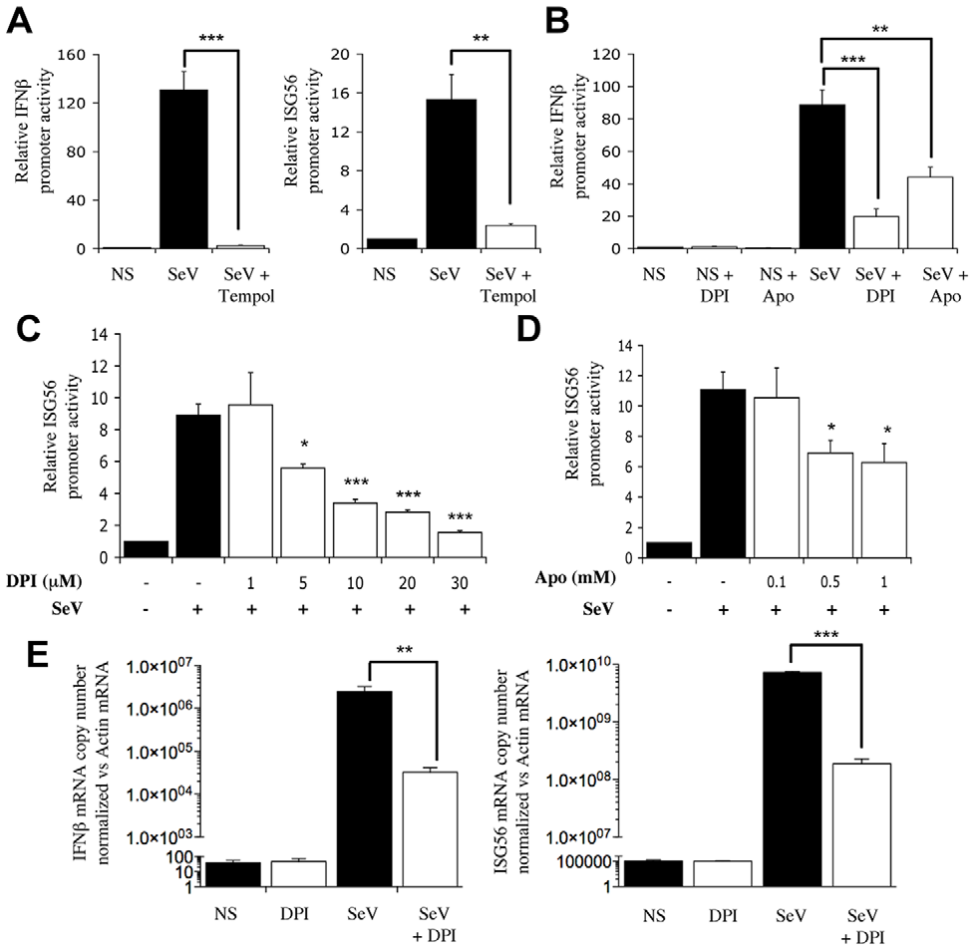
NADPH oxidase-derived ROS are required for SeV-induced *IFNβ* and *IFIT1* genes regulation. (**A–D**) A549 were transfected with the pRL-null renilla luciferase (internal control) and either the IFNβ-pGL3 or ISG56-pGL3 firefly luciferase reporter constructs. At 16h post-transfection, cells were pretreated with the indicated inhibitors (white bars), 3 mM Tempol, 1–30µM DPI (in B, a 10µM concentration was used) or 0.1–1mM Apo (in B, a 1mM concentration was used) or the corresponding vehicle (black bars), before being left unstimulated (NS) or infected with SeV (80 HAU/10^6^ cells) for 8h. Luciferase activities were expressed as fold activation over the corresponding NS condition after normalization with renilla luciferase activities. (**E**) A549 were treated with 30µM DPI (white bars) or the vehicle (black bars) and then left uninfected (NS) or infected with SeV (40 HAU/10^6^ cells) for 6h. Total RNA was extracted, subjected to reverse transcription and analyzed by real-time PCR using IFNβ-, ISG56- and actin-specific primers. mRNA levels are presented as absolute copy numbers of the target gene normalized versus actin mRNA used as a reference gene. (*, *p*<0.05; **, *p*<0.01; ***, *p*<0.001; mean ± SEM of triplicate experiments).

To start evaluating the potential implication of NOX-derived superoxide in IRF-3-mediated antiviral responses, the effect of antioxidants and pharmacological inhibitors on Sendai virus (SeV)-induced IFNβ- and ISG56-promoter activities was investigated in A549 cells. As shown in Figure 1A, SeV-induced IFNβ-promoter activity was significantly reduced in the presence of Tempol, a cell-permeable superoxide dismutase mimetic. Consistent with an implication of superoxide-dependent IRF-3 regulation, Tempol also inhibited the activity of the ISRE-containing ISG56-promoter in response to SeV (Figure 1A). Further analyses revealed that pretreatment with diphenyleneiodonium (DPI) or apocynin (Apo), two inhibitors classically used to target NADPH oxidase activities, also effectively inhibited SeV-induced IFNβ- (Figure 1B) and ISG56-promoter activities (Figure 1C and D) in a dose-dependent manner, while the same inhibitors did not similarly impact the activity of the unrelated pEF1 promoter (Figure S1 and Text S1).

To provide further evidence on whether NADPH oxidase-derived ROS are required for the regulation of IRF-3-dependent antiviral genes, the expression profile of *IFNβ* and *IFIT1* (encoding for ISG56) genes was monitored *in vivo* by real-time PCR during SeV infection in the absence or presence of DPI. While IFNβ and ISG56 mRNA levels were markedly induced following SeV infection, the mRNA levels exhibited 1.8 log and 1.6 log reduction, respectively, in the presence of DPI (Figure 1E). Altogether these results suggest that efficient *IFNβ* and *IFIT1* genes expression in response to SeV infection requires the production of ROS produced by a NADPH oxidase.

### Interference with NOX2 inhibits SeV-induced IFNβ- and ISG56-promoter activation

**Figure 2:**
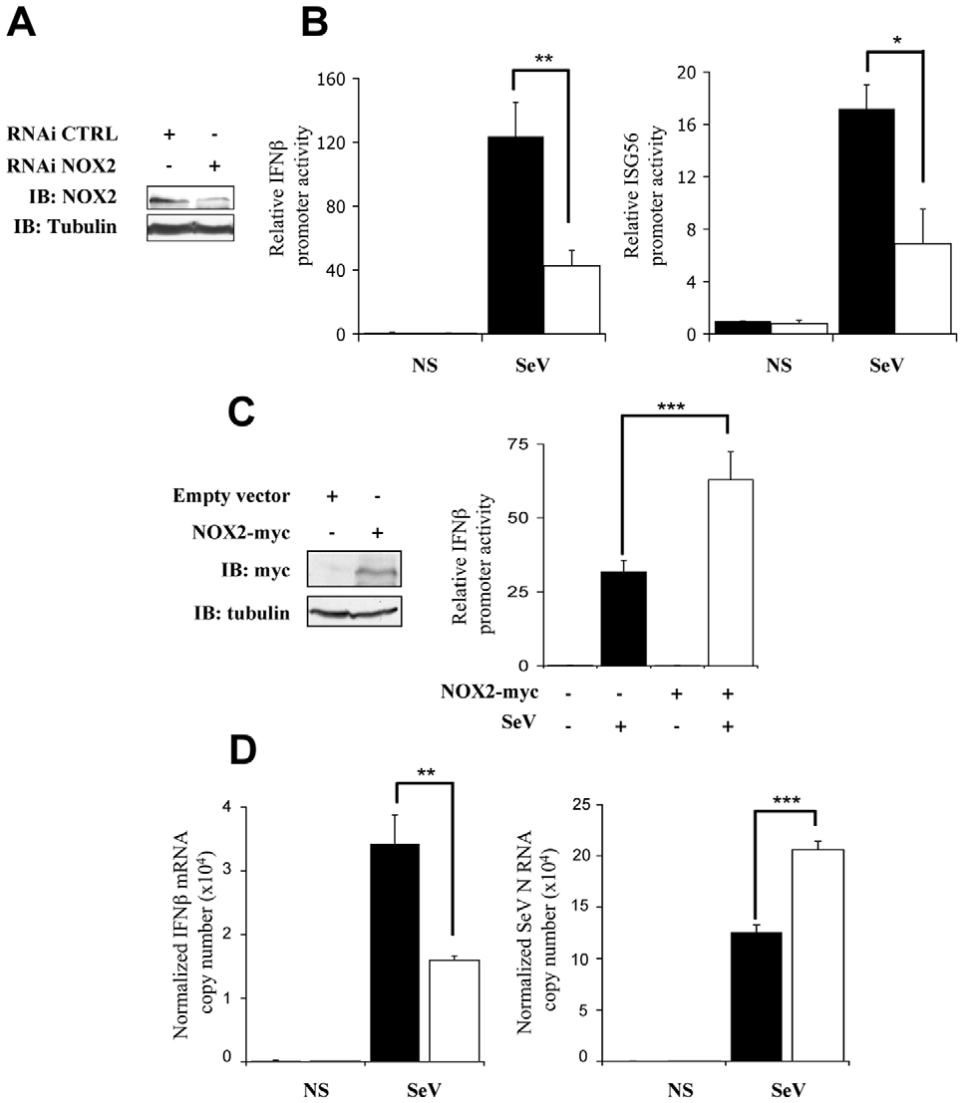
Interference with NOX2 expression inhibits SeV-induced *IFNβ* and *IFIT1* genes transactivation. (**A and B**) A549 cells were transfected with control- (CTRL; black bars) or NOX2-specific (white bars) RNAi oligonucleotides. (**A**) Efficiency of NOX2 knock down was monitored by immunoblot (IB) using anti-NOX2 antibodies. Anti-tubulin antibodies were used to control equal loading. (**B**) At 48h post-RNAi transfection, cells were further transfected with the IFNβ-pGL3 or ISG56-pGL3 firefly luciferase and the pRL-null renilla luciferase (internal control) reporter constructs and either left uninfected (NS) or infected with SeV (80 HAU/10^6^ cells). Luciferase activities were measured and expressed as described in Figure 1. (**C**) A549 cells were cotransfected with an empty control plasmid (black bars) or the myc-tagged-NOX2 (white bars) encoding plasmid and the IFNβ-pGL3 firefly luciferase and the pRL-null renilla luciferase (internal control) reporter constructs. At 16h post-transfection, cells were left unstimulated or infected with SeV for 8h and luciferase activities were measured and analyzed as described above. (**D**) Total RNA extracted from CTRL (black bars) and NOX2 RNAi (white bars)-transfected A549 either left uninfected (NS) or infected with SeV (5 HAU/10^6^ cells) for 5 hours were analyzed by reverse transcription and real-time PCR using IFNβ-, SeV N, and S9-specific primers. IFNβ mRNA levels are presented as absolute copy numbers normalized versus S9 mRNA used as a reference. SeV N fold expression values were determined using the ΔΔC_t_ method as described in Material and Methods.(*, *p*<0.05; **, *p*<0.01; ***, *p*<0.001; mean ± SEM of at least triplicate experiments).

To determine whether the observed antioxidant-mediated inhibition of SeV-induced *IFNβ* and *IFIT1* antiviral genes expression could be linked to an effect on NOX2, the effect of interference with NOX2 expression on their respective promoter activities was assessed. Immunoblot for NOX2 (Figure 2A) confirmed that NOX2 specific RNAi oligonucleotides significantly decreased NOX2 protein expression in A549, as previously validated and confirmed at the mRNA level [15], without affecting cellular viability (Figure S2 and Text S1). Reduction of NOX2 expression effectively altered the gene transactivation capacity of endogenous IRF-3. First, the stimulation of IFNß- and ISG56-promoter activities by SeV were dramatically decreased in NOX2-depleted cells compared to cells transfected with CTRL RNAi (Figure 2B). Second, ectopic expression of NOX2 significantly enhanced SeV-induced activation of the IFNβ promoter (Figure 2C). Additionally, to further establish the role of NOX2 *in vivo*, the expression of endogenous *IFNβ* was analyzed by real-time PCR following SeV infection of CTRL- and NOX2-RNAi-transfected cells. SeV-induced IFNβ mRNA level was significantly reduced by 53% in the absence of NOX2 as compared to control cells, while SeV replication quantified by real-time PCR analysis of the nucleocapside (N) RNA revealed a 1.6 fold increase (Figure 2D). Taken together, these results demonstrate that NOX2 contributes to the regulation of SeV-induced IFNβ- and ISG56-promoter activities, thereby suggesting that NOX2 is an essential component of the signaling pathway triggering IRF-3 activation following virus infection.

### SeV-induced IRF-3 activation is dependent on NOX2 and ROS production

**Figure 3:**
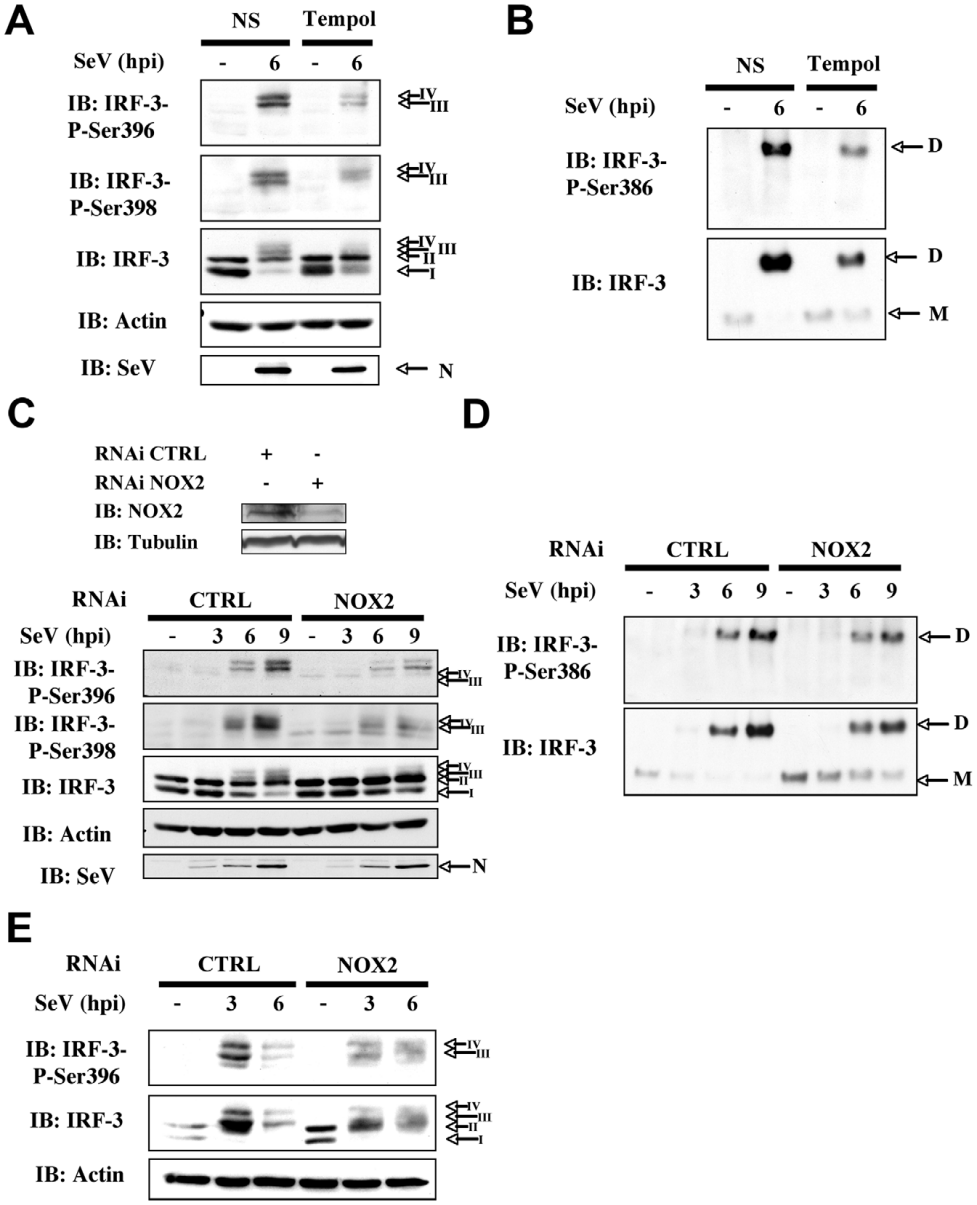
Knockdown of NOX2 expression impairs SeV-induced C-terminal IRF-3 phosphorylation and dimerization in A549 and human primary NHBE. (**A and B**), A549 were pretreated with 3mM Tempol or the corresponding vehicle for 1h. A549 cells (**C and D**) or NHBE cells (**E**) were transfected with control (CTRL) or NOX2 RNAi. (**A–E**) Cells were left uninfected or infected with SeV (10 HAU/10^6^ cells) for the indicated times. (**A, C** and **E**) WCE were analyzed by SDS-PAGE followed by immunoblot (IB) using anti-IRF3-Ser396 (IRF-3-P-Ser396) and anti-IRF3-Ser398 (IRF-3-P-Ser398) phosphospecific antibodies, anti-IRF-3 and anti SeV (the nucleocapsid N is shown) antibodies. Equal loading was controlled using anti-actin antibodies. (**B and D**), WCE analyzed in A and C were also resolved by native gel electrophoresis and revealed by immunoblot using anti-IRF3-Ser386 phosphospecific (IRF-3-P-Ser386) and anti-IRF-3 antibodies. M: monomer, D: dimer. Representative immunoblots of three different experiments are shown. hpi: hours post infection.

In uninfected cells, IRF-3 is predominantly present as two forms corresponding to an unphosphorylated (form I) and a N-terminus hypophosphorylated form (form II) [16]. The C-terminus of IRF-3 contains three clusters of virus-induced phosphoacceptor sites, Ser 385/386 (Cluster I), Ser 396/398 (Cluster II) and Ser 402/405 and Thr 404 (Cluster III) that are detected as two dimeric active forms (form III and IV) in SDS-PAGE [17], [18]. Thus, to provide further evidence that NOX2 and ROS are essential for IRF-3 activation, the effect of Tempol or NOX2 knock down through RNAi on IRF-3 phosphorylation and dimerization was analyzed. As shown in Figure 3A and B, SeV-induced phosphorylation of IRF-3 at Ser 396, and to a lesser extent at Ser 398, was significantly reduced in Tempol-treated compared to vehicle-treated cells. In the same line, formation of the active dimeric form of IRF-3, evaluated by native-PAGE, was also effectively impaired (Figure 3B). Phosphorylation at Ser 386 has previously been reported to correlate with IRF-3 in its dimeric form [18]. Accordingly, detection of Ser 386 was decreased in cells treated with Tempol (Figure 3B). Importantly, interference with NOX2 expression similarly inhibited SeV-induced IRF-3 Ser 396, 398 and 386 phosphorylation and dimerization (Figure 3C and D). Impairment of IRF-3 phosphorylation through interference with NOX2 was also confirmed in the context of SeV infection of primary cells, using normal human bronchial epithelial cells (NHBE) (Figure 3E). Altogether, these data demonstrate that NOX2 and ROS are essential for the efficient activation of IRF-3 during virus infection.

### NOX2 is required for IKK-related kinases activation in SeV infection

**Figure 4:**
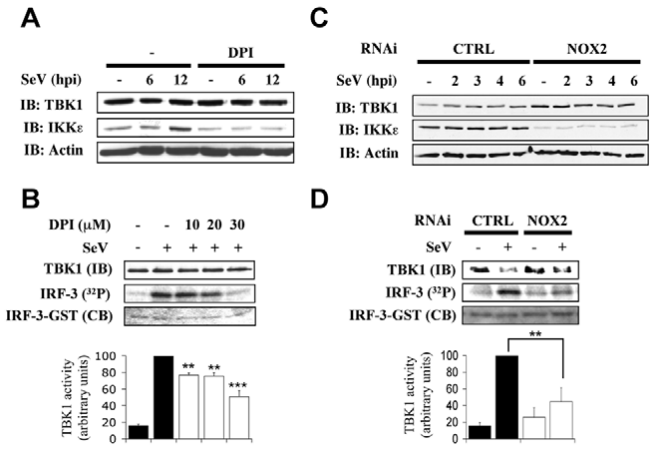
NOX2 is essential for IKKε expression and SeV-induced TBK1 activity. A549 were pretreated with the vehicle or the indicated concentrations of DPI (10–30µM) (**A and B**) or transfected with CTRL or NOX2 RNAi (**C and D**). Cells were then left uninfected or infected with SeV (40 HAU/10^6^ cells) and harvested at different hours post infections (hpi). (**A and C**), WCE were resolved by SDS-PAGE and analyzed by immunoblot (IB) using anti-TBK1, anti-IKKε and anti-actin antibodies. (**B and D**), TBK1 activity was monitored by *in vitro* kinase assay using GST-IRF-3-(aa387-427). Reactions were resolved by SDS-PAGE and IRF-3 substrate was detected by coomassie blue (CB) staining and radioactivity incorporation (^32^P). TBK1 activity was expressed as the ratio of radioactivity incorporation over the amount of immunoprecipitated kinase detected by immunoblot (IB) and quantified by densitometric analysis. Results are expressed as percentage of the activity measured after SeV infection in respective control cells. In B, black bars correspond to vehicle-treated cells, white bars corresponds to DPI-treated cells. In D, black bars correspond to CTRL RNAi-tranfected cells and white bars correspond to NOX2 RNAi-transfected cells.(**, *p*<0.01; ***, *p*<0.001; mean ± SEM of at least three independent experiments)

We and others have previously demonstrated that IKK-related kinases, IKKε and TBK1, are part of the kinase activity that is essential for IRF-3 phosphorylation and subsequent activation of IRF-3 in the context of virus infection [5], [6]. To start depicting the role of NOX2 in the upstream signaling pathways leading to IRF-3 activation, the role of NOX2 and ROS in SeV-induced TBK1/IKKε expression and activity was assessed. Initial analysis of kinases expression during SeV infection in the absence or presence of DPI or in CTRL RNAi vs NOX2 RNAi-transfected cells revealed that basal and SeV-induced expression of IKKε is dramatically decreased by DPI treatment or depletion of NOX2 expression (Figure 4A and C). Furthermore, quantification of SeV-induced TBK1 activity demonstrated that it is inhibited in a dose-dependent manner by DPI-treatment reaching around 52% inhibition at a concentration of 30 µM DPI compared to vehicle-treated cells (Figure 4B). Finally, in NOX2 RNAi-transfected cells, SeV-induced TBK1 activity was diminished by about 55% compared to the activity measured in CTRL RNAi-transfected cells (Figure 4D). These data provide important evidence for the requirement of NOX2 in the activation of IRF-3 kinases, TBK1 and IKKε, in response to SeV infection.

### Essential role of RIG-I in SeV-mediated IRF-3 activation in A549 cells

**Figure 5:**
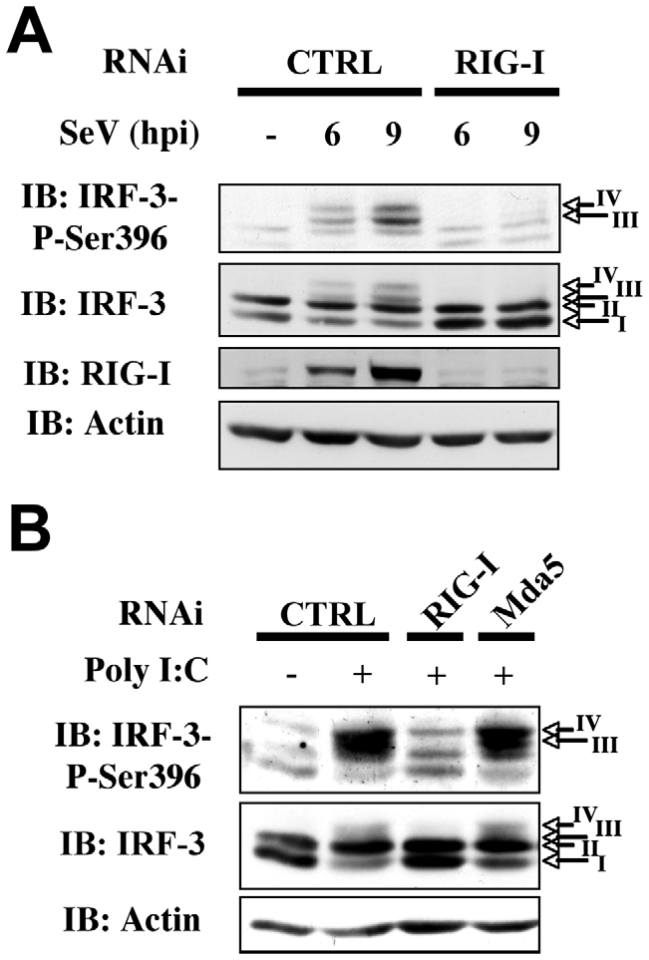
RIG-I is essential for SeV-induced and sheared poly I:C-induced IRF-3 activation. A549 were transfected with control (CTRL), RIG-I or Mda5 specific RNAi oligonucleotides as indicated. (**A**) A549 were further left unstimulated (NS) or infected with SeV (10HAU/10^6^ cells) for various hours post infection (hpi). (**B**) Cells were further transfected with sheared poly I:C or as control, subjected to the transfection reagent without poly I:C. WCE were resolved by SDS-PAGE and analyzed by immunoblot (IB) using anti-IRF-3-P-Ser396, anti-IRF-3, anti-RIG-I or anti-actin antibodies. Representative immunoblots of three different experiments are shown.

RLRs play unique and redundant roles in RNA virus recognition and appear to function in both cell- [19] and virus-specific manners [20], [21]. In order to further investigate the role of NOX2 in virus-mediated IRF-3 activation, we first thought to confirm, in our A549 model, the essential role of RIG-I in SeV recognition that was previously highlighted in embryonic fibroblasts (MEFs), lung fibroblasts, dendritic cells (DCs) and 293 cells [19], [20], [21], [22], [23]. RNAi specifically targeting RIG-I were used to determine the role of RIG-I in IRF-3 phosphorylation. As shown in Figure 5A, interference with RIG-I expression completely abolished IRF-3 Ser 396 phosphorylation following SeV infection, demonstrating that RIG-I is essential for downstream signaling to IRF-3 in the early time points following SeV infection in A549 cells.

Kato and collaborators recently reconciled previous conflicting results concerning the role of RIG-I and Mda5 in the recognition of poly I:C by showing that RIG-I and Mda5 selectively recognize short and long dsRNA, respectively [24]. Using lipid-mediated transfection of sheared poly I:C (see experimental procedures section for details on poly I:C preparation), we were able to specifically trigger IRF-3 Ser 396 phosphorylation by a RIG-I-dependent/Mda5-independent pathway (Figure 5B). Thus, activation of IRF-3 through the RIG-I-dependent pathway during SeV infection can be mimicked by poly I:C treatment in A549 cells.

### NOX2 and ROS are essential for RIG-I mediated IRF-3 phosphorylation and dimerization

**Figure 6:**
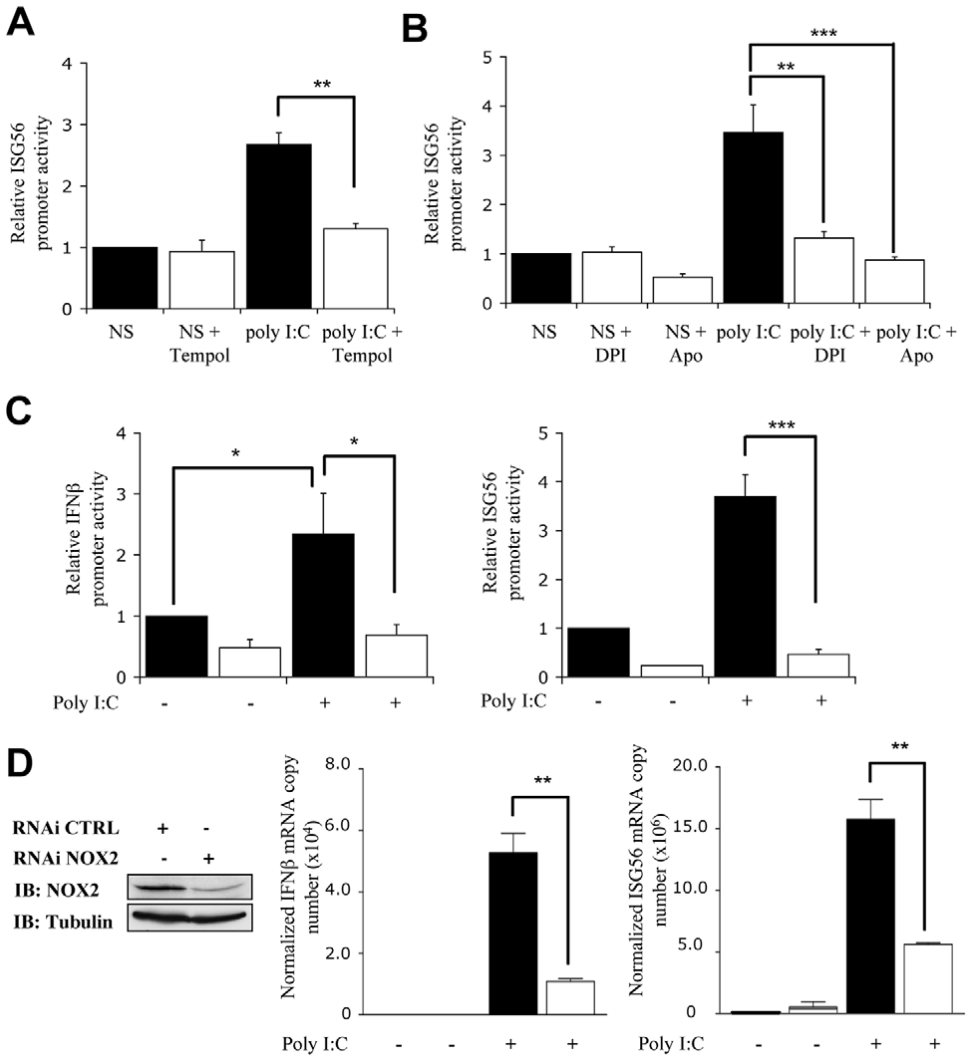
NOX2 is required for RIG-I-mediated regulation of *IFNβ* and *IFIT1* genes. (**A and B**) A549 were cotransfected with the ISG56-pGL3 firefly luciferase and the pRL-null renilla luciferase (internal control) reporter constructs. Cells were then pretreated with 3mM Tempol, 10µM DPI, 1mM Apo (white bars) or the corresponding vehicle (black bars) before being left unstimulated (NS) or transfected with poly I:C. (**C**) A549 were transfected with CTRL (black bars) or NOX2 (white bars) RNAi and were further cotransfected with the IFNβ- or ISG56-pGL3 and pRL-null reporter constructs and transfected with poly I:C for 8h. (**A–C**) Luciferase activities were measured and expressed as described in Figure 1. (*, *p*<0.05;**, *p*<0.01; ***, *p*<0.001; mean ± SEM of triplicates). (**D**) A549 were transfected with CTRL (black bars) or NOX2 specific (white bars) RNAi and left unstimulated or transfected with poly I:C for 3h. mRNA levels of IFNβ and ISG56 were analyzed by real-time PCR as described in Figure 1 after normalization to S9 mRNA used as a reference gene. NOX2 immunoblot was performed as described in Figure 2A. (*, *p*<0.05; ***, *p*<0.001; mean ± SEM of independent triplicates).

In order to further evaluate the potential role of NOX2 in the RIG-I-dependent signaling pathway, stimulation of A549 cells with poly I:C was performed in the absence or presence of antioxidants or NADPH oxidase inhibitors. As shown in Figure 6A and B, induction of the ISG56- promoter activity was significantly reduced when poly I:C treatment was performed in the presence of Tempol, DPI or Apo. Similarly, inhibition of NOX2 expression by RNAi also resulted in a dramatic diminution of the capacity of poly I:C to stimulate the IFNβ- and ISG56- promoters (Figure 6C). To further establish the role of NOX2 in IRF-3-dependent antiviral genes expression *in vivo*, the expression profile of *IFNβ* and *IFIT1* genes was analyzed by real-time PCR following poly I:C stimulation of CTRL and NOX2 RNAi-transfected cells. As illustrated in Figure 6D, poly I:C-induced IFNβ and ISG56 mRNA levels were significantly reduced in the absence of NOX2, as compared to control cells.

**Figure 7:**
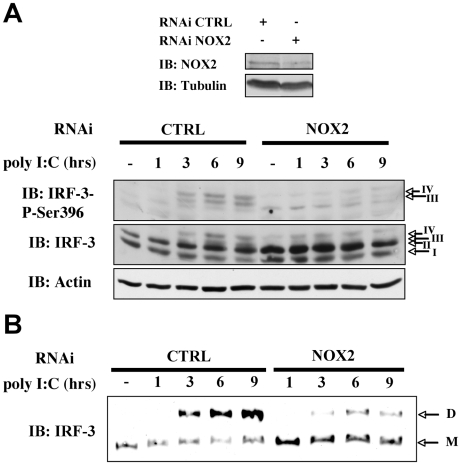
NOX2 depletion inhibits poly I:C-induced IRF-3 phosphorylation and dimerization. CTRL- and NOX2-RNAi-transfected A549 were subjected to mock or poly I:C transfection for the indicated times. (**A**) WCE were resolved by SDS-PAGE. Efficiency of NOX2 depletion by NOX2-RNAi was evaluated by immunoblot (IB) using tubulin detection as a loading control. On a separate gel, WCE were analyzed by IB using anti-IRF-3-P-Ser396, anti-IRF-3 and anti-actin antibodies. (**B**) WCE were resolved by native gel electrophoresis and immunoblotted with anti-IRF-3 antibodies. Representative immunoblots of three different experiments are shown. hpi: hours post infection. M: monomer, D: dimer.

Finally, to confirm that the observed inhibition of the poly I:C-induced IFNβ and ISG56 mRNA levels was mediated by alteration of IRF-3 activation, WCE derived from A549 transfected with CTRL or NOX2 RNAi and further stimulated with poly I:C, were analyzed for IRF-3 phosphorylation. In cells where NOX2 expression is significantly downregulated by RNAi, poly I:C-induced IRF-3 phosphorylation at Ser396 was barely detectable, while it was significantly induced in CTRL RNAi transfected cells (Figure 7A and B). Consistently, poly I:C-induced IRF-3 dimerization was strongly impaired in NOX2 RNAi vs CTRL RNAi transfected cells (Figure 7C). Altogether these results provide strong evidence that NOX2 and ROS are essential for RIG-I-induced, IRF-3-mediated antiviral gene transcription.

### Downregulation of NOX2 and superoxide scavenging inhibit MAVS expression

**Figure 8:**
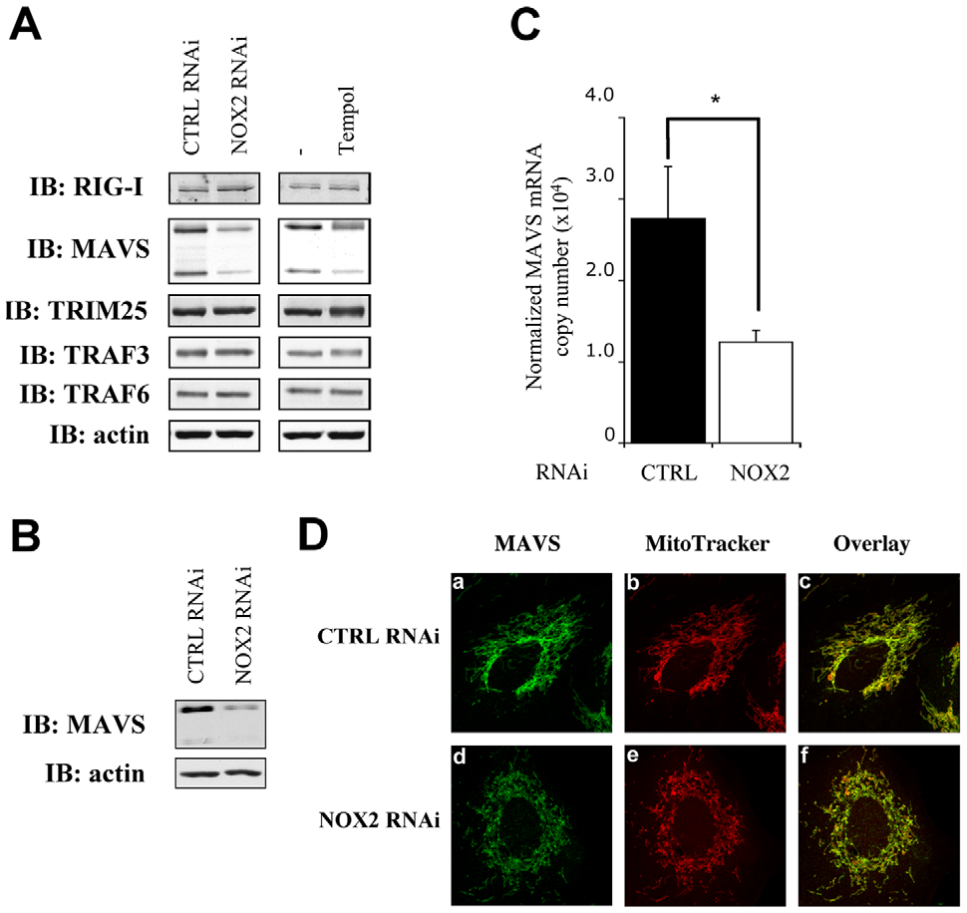
NOX2 downregulation or ROS scavenging diminishes MAVS mRNA expression without affecting its subcellular localization. (**A, B**) WCE derived from A549 (**A**) and NHBE (**B**) cells transfected with control (CTRL) or NOX2 RNAi or treated with vehicle or 3mM Tempol were analyzed by immunoblot using anti-RIG-I, anti-MAVS, anti-TRIM25, anti-TRAF3, anti-TRAF6 and anti-actin antibodies. MAVS was detected as two different splice variants as described in [66]. Representative immunoblots of at least three different experiments are shown. (**C**) Total RNA extracted from A549 transfected with CTRL and NOX2 RNAi were analyzed by real time PCR as described in Figure 1. MAVS mRNA levels are expressed as absolute values after normalization to S9 mRNA used as a reference gene. (*, *p*<0.05; mean ± SEM of independent triplicates). (**D**) A549 cells transfected with CTRL and NOX2 RNAi were fixed and mitochondria were stained using Mitotracker (red; panel b and e). After permeabilization, MAVS was stained using anti MAVS antibodies and Alexa488-secondary antibodies (green; panel a and d). Images were overlaid to observe colocalization (panel c and f). Images are representative of 2 experiments performed in triplicate.

Based on the observation that NOX2 downregulation and antioxidant treatments reduced the ability of cells to mount an efficient RIG-I mediated antiviral response through inhibition of IRF-3 phosphorylation, the regulation of signaling molecules upstream of IRF3 kinases was evaluated. Analysis of the expression of known upstream signaling molecules by immunoblot revealed that MAVS level is dramatically reduced in NOX2 vs CTRL RNAi-transfected A549 (Figure 8A) and NHBE (Figure 8B) cells. On the other hand, expression of RIG-I, of the ubiquitin ligase TRIM25 that is known to regulate RIG-I activity [25], and of the ubiquitin ligase TRAF3 that interacts with MAVS to trigger virus-induced IRF-3 activation were similar in both conditions. Moreover, expression of the TRAF6 ubiquitin ligase involved in MAVS-mediated NF-κB activation, which is also placed under NOX2 control [15], was also found to be equal in both conditions (Figure 8A). A specific reduction of MAVS protein level was also observed in Tempol-treated A549 cells compared to control cells (Figure 8A). Further analysis of MAVS expression by real-time PCR demonstrated that NOX2 downregulation by RNAi resulted in a 55% reduction of MAVS mRNA compared with control cells transfected with CTRL RNAi (Figure 8C). As previous reports highlighted a key role of MAVS localization in the mitochondria outer membrane to its function [26], and anticipating that NOX2 might regulate MAVS at different levels, the localization of the remaining amount of MAVS was also visualized by confocal microscopy. MAVS staining colocalized with mitochondrial marker in both control and NOX2-depleted A549 cells (Figure 8D), thus excluding an effect of NOX2 on MAVS function through modulation of its subcellular localization. Taken together these data demonstrate that in AEC, NOX2 and ROS promotes MAVS mRNA expression.

## Discussion

NOX enzymes are the main source of deliberate cellular ROS production in response to various stimuli. Numerous studies over the past years have increasingly clarified the function of NOX in various biological processes, including cell proliferation, apoptosis, proinflammatory response and host defense, notably through their role as cellular switches that regulate signal transduction pathways [7], [8]. The importance of NOX enzymes in the innate host defense is exemplified by the role of NOX2 in the generation of high amount of ROS, known as oxidative burst, in phagocytic cells as part of their armory of anti-bacterial mechanisms [27]. In this study, we reveal a novel essential function of NOX2-derived superoxide in the innate immune antiviral response triggered following recognition of invading virus by the RIG-I cytoplasmic sentinel. Our data demonstrate that efficient IRF-3 activation and downstream antiviral genes, *IFIT1* and *IFNβ*, expression in response to SeV or RIG-I stimulation are impaired by NOX2 knock down, pharmacological inhibition of NOX or treatment with superoxide dismutase mimetic.

Few reports previously identified a role of NOX2 in other aspects of the cellular response to virus infections. NOX2 was recently shown to mediate HIV Tat-induced JNK activation and cytoskeletal rearrangement in HUVEC [28]. Moreover, *Paramyxoviridae* virus-induced NF-κB activation and downstream proinflammatory cytokines production in AEC were shown to be dependent on NOX2 [15]. Reduced inflammation of lung parenchyma has also been noticed during influenza infection in mice lacking NOX2 murine homolog [29]. However, this study does not distinguish between a role of NOX2 in phagocytes recruited to the lung parenchyma from a potential role of NOX2 in non-phagocytic cells. Earlier reports suggested NOX-dependent IRF-3 activation in response to Respiratory Syncitial Virus (RSV), but this conclusion was only based on an inhibitory effect of DPI or Apo treatment [30]. Based on the previous demonstration that RSV-induced IRF-3 activation is triggered by a RIG-I-dependent recognition mechanism [31] and our recent observation that NOX2 is involved in RSV-mediated NF-κB activation in AEC [15], the role of NOX2 in RIG-I-mediated activation of IRF-3, presented in this study, provides a likely mechanism for this yet unexplained redox-dependent activation of IRF-3 during RSV infection.

Beside RLH receptors, viral nucleic acids are also sensed by members of the Toll-like receptor (TLR) family, including TLR-3 that binds extracellular or endosomal dsRNA [1]. Exogenous H_2_O_2_ treatment was recently shown to enhance TLR-3 mediated NF-κB, but not IRF-3, activation [32]. This result suggests that redox-dependent regulation of IRF-3 following recognition of viral RNA is not a universal mechanism, but depends on the PRR engaged following virus infection. However, it is also important to consider that the use of exogenous H_2_O_2_ constitute a major difference with our study. Indeed, not only the subtype of ROS, but also the localization of the ROS signal at specific subcellular compartment, is considered essential for activating specific redox signaling events [33], [34]. Thus, the effect of exogenously added H_2_O_2_ most likely differs from the role of endogenous NOX2-dependent superoxide studied here. Interestingly, IRF3 activation following TLR-4 stimulation with LPS stimulation in U373/CD14 appears to be dependent on the activity of another member of the NOX family, NOX4 [35]. In this study, NOX4 appears to play a role in an ASK1/p38 axis that leads to IRF-3 nuclear accumulation. However, whether NOX4 is also involved in the TBK1/IKKε pathway that was shown to be involved in LPS-induced IRF-3 activation in macrophages [36] has not yet been investigated. In the present study, a role of NOX4 in the RIG-I-mediated IRF-3 activation was excluded based on the absence of detection of NOX4 expression in the A549 cell model used, as previously described [15].

Recently, a role of ROS in RLR signaling was documented in Atg5^−/−^ MEF cells that are defective in autophagy process. ROS associated with the accumulation of dysfunctional mitochondria in these cells enhanced RLR-mediated cytokines production [37]. In the same study, rotenone treatment, which artificially induces accumulation of mitochondria-associated ROS, was sufficient to enhance RLR signaling [37]. However, it is not yet clear how this ROS-dependent mechanism is involved in RLR signaling regulation in normal cells exhibiting functional autophagy and if the effect of mitochondria-associated ROS is associated with increased IRF-3 activation. This production of ROS at a non-physiological level is considered harmful, and therefore might represent more a deleterious mechanism involved in virus pathogenicity than a physiological regulation of RLR signaling as illustrated by our results. Other studies also presented evidence of a cross-talk between NOX2 and mitochondria-associated ROS in the regulation of signaling cascades. In human umbilical vein endothelial cells and human alveolar macrophages, TNFα-induced NF-κB activation was shown to be dependent on both NOX2 and the mitochondrial respiratory chain activity [38], [39]. Thus, one may not exclude at this point that both the NOX2-dependent and a mitochondria-associated mechanism might cooperate in the regulation of RIG-I-mediated activation of the antiviral response.

Since NOX2 knock down alters phosphorylation of multiple phosphoacceptor sites, including Ser 386, Ser 396 and Ser 398 in the C-terminal clusters of IRF-3, it likely plays a role in several pathways converging to IRF-3 activation downstream of RIG-I. Our data demonstrate that NOX2 is required for TBK1 catalytic activation and IKKε expression. Recent studies strongly suggest that TBK1 and IKKε specifically phosphorylate Ser 402 and Ser 396 of IRF-3 [40], [41], [42], thereby implying that yet unidentified kinases that are responsible for the phosphorylation of other critical phosphoacceptor sites might also be controlled by NOX2. Recently, Protein kinase C-α [43], PI3 kinase [44], IKKα [45] and JNK [46], were shown to be involved in IRF-3 phosphorylation, but whether these kinases directly target IRF-3, and if so, on which specific phosphoacceptor sites, has not yet been established.

The observation that NOX2 knock down and antioxidant treatment abrogated RIG-I mediated IRF-3 phosphorylation raised the question about the identity of the molecular target(s) in the RIG-I-induced signaling cascade. Our data demonstrate that in AEC, NOX2 is essential for expression of the MAVS adaptor, which acts as a central platform to catalyze the formation of the mito-signalosome containing, among other signaling molecules, RIG-I and TRAFs involved in the antiviral cascade [47]. ROS and NADPH oxidases are known to regulate mRNA expression by several means, including regulation of redox-sensitive transcription factors, such as NF-κB and AP-1 [48], [49] and modulation of mRNA stability as reported elsewhere for TLR-4, IL-1 or p53 mRNA [26], [50], [51]. Interestingly, NOX2 was specifically shown to regulate cell cycle via induction of p21^cip1^ mRNA expression in endothelial cells following nutrient deprivation [52]. The transcriptional and post-transcriptional mechanisms involved in MAVS mRNA expression have not yet been elucidated. Further studies are required to identify these mechanisms and uncover how NOX2 is involved in their regulation.

Function of NOX2-derived ROS under basal conditions was previously documented in other non-phagocytic cells [53], [54], [55]. Particularly, an active NOX2-containing NADPH oxidase is well documented to contribute to endothelial cells proliferation [53], [54]. It is worth mentioning that during the course of our study, Takemura and Collaborators identified a role of NOX2 in the regulation of basal and EGF-induced ENac channel activity in alveolar epithelial cells [56]. Our data describe the essential role of NOX2 and ROS in basal MAVS mRNA and protein expression. However, during virus infection, MAVS is proteolysed by a proteasome-dependent PCBP2/AIP4 axis [57]. Thus, it is an interesting working hypothesis that NOX2-dependent mechanism might permit expression of MAVS during virus infection to regenerate the pool of available MAVS in the cell to mount a sustained antiviral response. Studies are underway to characterize in detail basal and virus-induced NOX2 activity. To date, NOX2 subcellular localization in non-phagocytic cells appears to vary from cell type to cell type, being either at the plasma membrane, in the endosomes responsible for early receptor-mediated signaling, known as “redoxisomes”, or in the perinuclear region [58]. An interesting aspect of these studies will be to determine the localization of NOX2 and ROS production involved in MAVS expression, as this is now considered an important aspect in the specificity of redox-dependent functions [59].

## Materials and Methods

### Reagents

DPI, DMSO and BSA were purchased from Sigma-Aldrich. Apo was purchased from Calbiochem. Tempol was from Biomol International. Oligonucleotide primers were purchased from Invitrogen (Carlsbad, USA) or Alpha DNA (Montreal, Canada). RNAi oligonucleotides were from Dharmacon. Target sequences of the RNAi used against the different genes are as followed: CTRL, ^5′^cauagcguccuugatcaca^3′^ ; NOX2, ^5′^gaagacaacuggacaggaa^3′^ ; RIG-I, ^5′^aacgauuccaucacuauccau^3′^ ; Mda5, ^5′^ggugaaggagcagauucag^3′^ .

### Plasmids

The pRL-null reporter plasmid was obtained from Promega. ISG56-pGL3 and IFNβ-pGL3 luciferase reporter constructs and GST-IRF-3(aa387-427)-pGex-KG expression plasmid were previously described [4], [5]. Plasmids used to establish Real-Time PCR standard curves were generated by cloning of the PCR-amplified +208 to +706nt fragment of the IFNβ transcript (NM_002176), the +335 to +1319nt fragment of the IFIT1 transcript (NM_001548), the +748 to +980nt fragment of the β-actin transcript (NM_001101) and the +57 to +652nt fragment of the ribosomal S9 transcript (NM_001013) into the pCR2.1-TOPO using *Eco*RI. The pCS2-myc-NOX2 construct encoding myc-tagged human NOX2 was a kind gift from Dr. Shah, King's College London [60]. The pcDNA3.1-myc-MAVS construct was previously described [61].

### Cell culture and infections

A549 cells (ATCC) were cultured in F12 Nutrient Mixture (Ham) medium (Gibco) supplemented with 10% heat-inactivated Fetal Bovine Serum (HI-FBS, Gibco) and 1% L-Glutamine (Gibco). Normal human bronchial epithelial cells (NHBE) were obtained from Lonza, cultured in BEGM medium (Lonza) and used between passage 2 and 4. Sendai virus Cantell strain was obtained from Charles River Laboratories. Infection of subconfluent cells was performed at 10–80HAU/10^6^ cells depending on the experiments for the indicated times. Where indicated, DPI, Apocynin or Tempol (or the corresponding vehicle) were added 1h before infection in serum free medium (SFM). Infection was pursued for 2h in SFM before addition of HI-FBS.

### Stimulation with poly I:C

The synthetic analog of dsRNA, polyinosine-polycytidylic acid (poly I:C) (InvivoGen, San Diego CA) was resuspended at 10 mg/ml in sterile PBS and annealed by heating at 56°C for 30 min before cooling down at RT until it reached 20°C. Before use, poly I:C was diluted to 1mg/mL with ice-cold PBS and sheared using a 26G syringe. Cells were then transfected using Lipofectamine 2000 (Invitrogen) according to the manufacturer's instructions.

### RNAi oligonucleotides and plasmid transfections and luciferase assays

RNAi oligonucleotides and plasmid transfections were performed using the oligofectamine reagent (Invitrogen) and the *Trans*IT-LT1 Transfection Reagent (Mirus), respectively, and luciferase assays were performed using the Dual Luciferase reporter assay (Promega) as previously described in [15].

### Immunoblot analysis

Whole cell extracts (WCE) were prepared on ice in Nonidet P-40 (Igepal, SIGMA) lysis buffer [15] and quantified using the Bio-Rad Protein Assay (Biorad, Hercules CA). 30µg were subjected to SDS-PAGE electrophoresis followed by immunoblot analysis using anti-IRF-3-phosphoSer396 [62], anti-IRF-3-phosphoSer398 [36], anti-IRF-3 (Active Motif), anti-TBK1 (Imgenex), anti-IKKε (eBioscience), anti-MAVS (Alexis Biochemicals), anti-RIG-I (Alexis Biochemicals), anti-TRAF3 (santa-cruz), anti-TRAF6 (santa-cruz), anti-TRIM25 (BD transduction laboratories), anti-actin (Chemicon International) and anti-SeV (obtained from Dr. J. Hiscott, McGill University, Montreal, Canada) antibodies diluted in PBS containing 0.5% Tween and either 5% nonfat dry milk or 5% BSA.

For NOX2 detection, A549 were scraped directly in 125mM Tris-HCl (pH 6.8), 10% glycerol, 2% SDS, 0.1 M DTT buffer containing 10µg/ml leupeptin, 20µg/ml aprotinin and 1µM pepstatin. Lysis was pursued at RT for 15 min before sonication. After 10 min at 70°C, samples were quantified using RC-DC protein quantification assay (BioRad). 150µg of lysate proteins were resolved by SDS-PAGE and analyzed by immunoblot with anti-gp91phox-Cter (obtained from Dr. Dagher and Dr. Brandolin, CEA-Grenoble, Grenoble, France) and anti-tubulin (Santa-Cruz) antibodies.

Immunoreactive bands were visualized by enhanced chemiluminescence using the Western Lightning Chemiluminescence Reagent Plus (Perkin Elmer Life Sciences). In between phosphospecific- and anti-IRF-3-antibodies, the membrane was stripped in 0.2% SDS, 62.5 mM Tris-HCl pH 6.8, 0.1 mM β-mercaptoethanol for 20 minutes at 50°C.

### Dimerization assay

Native-PAGE was conducted as described previously [63] using 8µg WCE prepared as described above. Immunoblot detection of IRF-3 was performed using anti-IRF-3-phospho-386 (1/200, IBL) or anti-IRF-3 (Active Motif) antibodies.

### *In vitro* kinase assays

*In vitro* kinase assays were conducted as described previously [42], using 80µg of WCE immunoprecipitated using 1µg of TBK1 antibodies (obtained from Dr. T. Maniatis, Harvard, USA) and 1µg of recombinant GST-IRF-3 (aa387-427) protein produced in BL21(DE3)plysS following IPTG stimulation as previously described [64]. After resolution by SDS-PAGE, IRF-3-GST was detected by coomassie blue staining of the lower part of the gel and radioactivity incorporation (^32^P) was quantified using a Typhoon Trio apparatus (Amersham Biosciences). The upper part of the gel was transferred to nitrocellulose membrane and TBK1 was detected using anti-TBK1 antibodies (Imgenex).

### RNA extraction and real-time PCR

Total RNA was prepared using the RNAqueous-96 Isolation Kit (Ambion) following the manufacturer's instructions without the included DNase1 treatment step. Total RNA (1µg) was subjected to reverse transcription using the QuantiTect Reverse Transcription Kit (Qiagen), which includes a genomic DNA removal step. PCR amplifications were performed using the QuantiTect SYBR Green Kit (Qiagen) or the Fast start SYBR Green Kit (Roche) in the presence of 0.4µM of ISG56-, IFNβ-, β-actin- or –S9 specific primers. Absence of genomic DNA contamination was analyzed using a reaction without reverse transcriptase. Sequences of primer used are as follows: - ISG56, S: gcccagacttacctggacaa, AS: ggttttcagggtccacttca- IFNß, S: gaactttgacatccctgaggagattaagcagc, AS: gttccttaggatttccactctgactatggtcc- SeV N, S: agtatgggaggaccacagaatgg, AS: ccttcaccaacacaatccagacc- MAVS, S: ggtgccatccaaagtgcctacta, AS: cagcacgccaggcttactca- S9, S: cgtctcgaccaagagctga, AS: ggtccttctcatcaagcgtc- Actin, S: acaatgagctgctggtggct; AS: gatggccacagtgtgggtga. Detection was performed on a Rotor-Gene 3000 Real Time Thermal Cycler (Corbett Research). For *ISG56*, *IFNβ*, actin and *S9*, standard curves were obtained using amplification of serial dilutions of pCR2.1-TOPO-ISG56, -IFNβ, -β-actin and –S9 plasmids. ISG56 and IFNβ data represent absolute mRNA copy numbers normalized to β-actin or S9 used as a reference gene. For SeV N expression, standard curves and PCR efficiencies were obtained using serial dilutions of cDNA prepared from positive control infected cells and data are presented as relative fold expression versus uninfected sample after normalization to S9. Relative fold expression values were determined applying the ΔΔC_t_ method [65].

### Confocal immunofluorescence imaging

A549 cells were grown and transfected with RNAi oligonucleotides on glass coverslips. At 48h post-transfection, mitochondria were visualized with 100nM Mitotracker Red CMX ROS (Invitrogen) applied to cells for 30 minutes at 37C. Cells were subsequently washed, fixed with 3.7% formaldehyde for 15 minutes at 37C and permeabilized with 0.2% triton X-100. Cells were then blocked in 10% goat serum before incubation for 3h with anti-MAVS (Alexis Biochemicals) diluted in PBS containing 3% BSA. After washing, cells were incubated for 1h with anti-rabbit Alexa488 secondary antibody (Invitrogen) diluted in PBS containing 3% BSA. Cells were then washed and mounted with ProLong Antifade reagent (Invitrogen). Single plane images were acquired with a Leica SP5 confocal microscope equipped with 63× (1.7 NA) oil objective with a digital zoom of 2×.

### Statistical analyses

Data are presented as the mean ± standard error of the mean (SEM). Statistical significance for comparison of two means was assessed by an unpaired Student's *t* test. For dose-dependent experiments or multiple inhibitor studies, a one-way analysis of variance (ANOVA) test was used followed by a Dunnett post-test. Analyses were performed using the Prism 5 software (GraphPad). Statistical relevance was evaluated using the following *p* values: *p*<0.05 (*), *p*<0,01 (**) or *p*<0,001 (***).

### Accession numbers

Please see Table 1 for accession numbers.

**Table 1:** Accession numbers.

	mRNA	Protein
IRF-3	NM_001571.4	NP_001562.1
RIG-I	NM_014314.3	NP_055129.2
MAVS	NM_020746.3	NP_065797.2
TBK1	NM_013254.2	NP_037386.1
IKKε	NM_014002.2	NP_054721.1
TRIM25	NM_005082.4	NP_005073.2
TRAF3	NM_003300.2	NP_003291.2
TRAF6	NM_004620.2	NP_004611.1

## Supporting Information

Text S1 Supporting information
(0.03 MB DOC)

Figure S1 Effect of Tempol, Apocynin and DPI on the pEF1 unrelated promoter. A549 were transfected with the pRL-null renilla luciferase (internal control) and either the pEF1-Luc firefly luciferase reporter constructs. At 16h post-transfection, cells were pretreated with the following inhibitors (white bars), 3 mM Tempol, 10µM DPI or 1mM Apo or the corresponding vehicle (black bars), before being left unstimulated (NS) or infected with SeV (80 HAU/10^6^ cells). Luciferase activities were normalized over renilla luciferase activities (mean+/− SEM of triplicate experiments).
(0.08 MB TIF)

Figure S2 Cell viability of CTRL- vs NOX2-RNAi transfected A549. A549 were transfected as described with control (CTRL) or NOX2 specific RNAi. At 48h post-transfection, viable and non-viable cells were quantified by trypan blue exclusion assay. Data are expressed as percent over the total cell number (mean+/− SEM of triplicate experiments).
(0.09 MB TIF)
