# Supplementary material for: Requirement of NOX2 and Reactive Oxygen Species for Efficient RIG-I-Mediated Antiviral Response through Regulation of MAVS Expression
Source: PLoS Pathog. 2010 Jun 3;6(6):e1000930. doi: 10.1371/journal.ppat.1000930 (PMC2880583; doi:10.1371/journal.ppat.1000930)
Supplement: Text S1 — Supporting information (0.03 MB DOC) [file ppat.1000930.s001.doc]

**Supplemental Material and Methods.**

*Plasmids*

The pEF1-Luc construct contains the pEF1promoter cloned upstream of the Luciferase encoding open reading frame in a pcDNA3.1 backbone. The CMV promoter of the original pcDNA3.1 plasmid was replaced by the pEF1 promoter and the luciferase open reading frame was cloned in the modified MCS.

*Cell viability quantification.*

Viability of RNAi-transfected A549 cells was determined using trypan blue exclusion assay at 48h post-transfection.

**Supplemental figure legends**

**Figure S1. Effect of Tempol, Apocynin and DPI on the pEF1 unrelated promoter.**

A549 were transfected with the pRL-null renilla luciferase (internal control) and either the pEF1-Luc firefly luciferase reporter constructs. At 16h post-transfection, cells were pretreated with the following inhibitors (white bars), 3 mM Tempol, 10μM DPI or 1mM Apo or the corresponding vehicle (black bars), before being left unstimulated (NS) or infected with SeV (80 HAU/10^6^ cells). Luciferase activities were normalized over renilla luciferase activities (mean+/- SEM of triplicate experiments).

**Figure S2. Cell viability of CTRL- vs NOX2-RNAi transfected A549.**

A549 were transfected as described with control (CTRL) or NOX2 specific RNAi. At 48h post-transfection, viable and non-viable cells were quantified by trypan blue exclusion assay. Data are expressed as percent over the total cell number (mean+/- SEM of triplicate experiments).
