# Supplementary figures and images for: Requirement of NOX2 and Reactive Oxygen Species for Efficient RIG-I-Mediated Antiviral Response through Regulation of MAVS Expression
Source: PLoS Pathog. 2010 Jun 3;6(6):e1000930. doi: 10.1371/journal.ppat.1000930 (PMC2880583; doi:10.1371/journal.ppat.1000930)

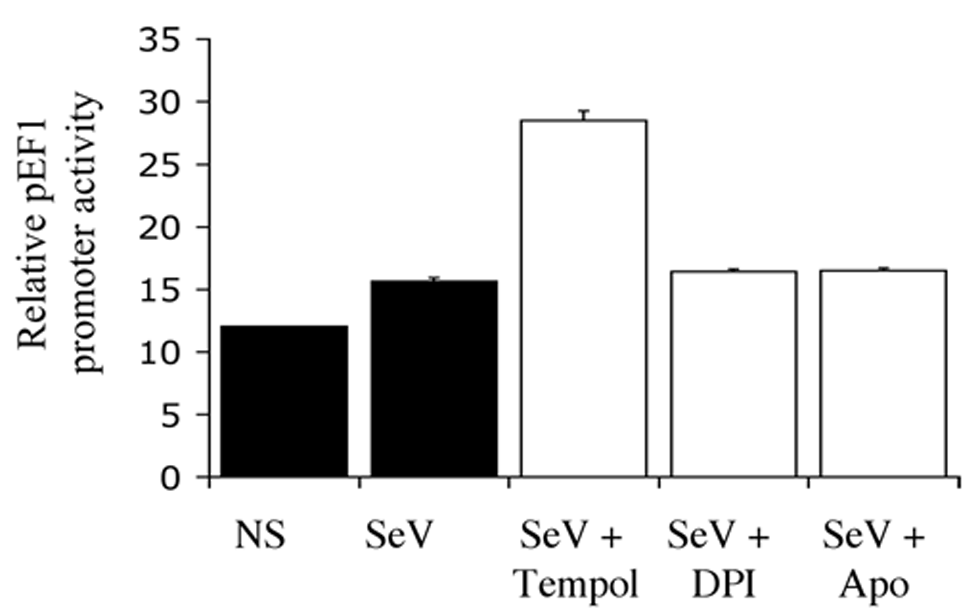

Supplement: Figure S1 — Effect of Tempol, Apocynin and DPI on the pEF1 unrelated promoter. A549 were transfected with the pRL-null renilla luciferase (internal control) and either the pEF1-Luc firefly luciferase reporter constructs. At 16h post-transfection, cells were pretreated with the following inhibitors (white bars), 3 mM Tempol, 10µM DPI or 1mM Apo or the corresponding vehicle (black bars), before being left unstimulated (NS) or infected with SeV (80 HAU/106 cells). Luciferase activities were normalized over renilla luciferase activities (mean+/− SEM of triplicate experiments). (0.08 MB TIF) [file ppat.1000930.s002.tif]

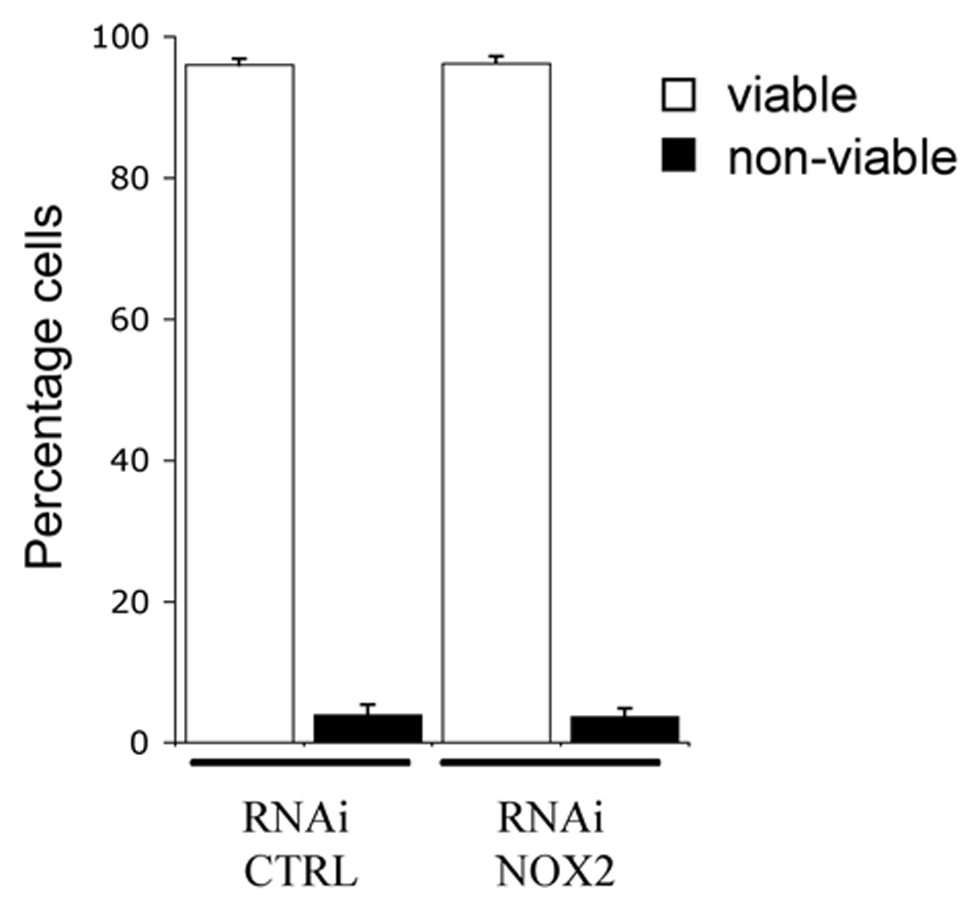

Supplement: Figure S2 — Cell viability of CTRL- vs NOX2-RNAi transfected A549. A549 were transfected as described with control (CTRL) or NOX2 specific RNAi. At 48h post-transfection, viable and non-viable cells were quantified by trypan blue exclusion assay. Data are expressed as percent over the total cell number (mean+/− SEM of triplicate experiments). (0.09 MB TIF) [file ppat.1000930.s003.tif]
